# Laboratory strains of *Escherichia coli* K-12: things are seldom what they seem

**DOI:** 10.1099/mgen.0.000922

**Published:** 2023-02-06

**Authors:** Douglas F. Browning, Jon L. Hobman, Stephen J. W. Busby

**Affiliations:** ^1^​ School of Biosciences, College of Health and Life Sciences, Aston University, Aston Triangle, Birmingham B4 7ET, UK; ^2^​ School of Biosciences, University of Nottingham, Sutton Bonington Campus, Sutton Bonington, Loughborough LE12 5RD, UK; ^3^​ Institute of Microbiology and Infection, School of Biosciences, University of Birmingham, Birmingham B15 2TT, UK

**Keywords:** bacteriophage lambda, *Escherichia coli *K-12, F plasmid, genomic analysis, laboratory-based evolution

## Abstract

*
Escherichia coli* K-12 was originally isolated 100 years ago and since then it has become an invaluable model organism and a cornerstone of molecular biology research. However, despite its pedigree, since its initial isolation *E. coli* K-12 has been repeatedly cultured, passaged and mutagenized, resulting in an organism that carries many genetic changes. To understand more about this important model organism, we have sequenced the genomes of two ancestral K-12 strains, WG1 and EMG2, considered to be the progenitors of many key laboratory strains. Our analysis confirms that these strains still carry genetic elements such as bacteriophage lambda (λ) and the F plasmid, but also indicates that they have undergone extensive laboratory-based evolution. Thus, scrutinizing the genomes of ancestral *E. coli* K-12 strains leads us to examine whether *E. coli* K-12 is a sufficiently robust model organism for 21st century microbiology.

## Data Summary

Figs S1–S14 and File S1 are available with the online version of this article. All genome sequence data and assemblies have been deposited in National Center for Biotechnology Information (NCBI) GenBank under BioProject ID PRJNA848777. The assembled and annotated genomes of WG1 and EMG2 have been deposited with the accession numbers CP099590 and CP099591 (WG1) and CP099588 and CP099589 (EMG2).

**Impact Statement**

Since its isolation in 1922, *Escherichia coli* K-12 has become arguably the premier model organism for contemporary science. Adoption of *E. coli* K-12 by many microbiologists across the globe has meant that it has a complex pedigree and, although many *E. coli* K-12 strains have been sequenced, little is known about the early versions of K-12, which still carry the F plasmid and bacteriophage λ. To understand more about this important model organism, we have sequenced two ancestral K-12 strains, WG1 and EMG2, which are considered to be the progenitors of many of the laboratory strains used today.

## Introduction

**Fig. 1.**
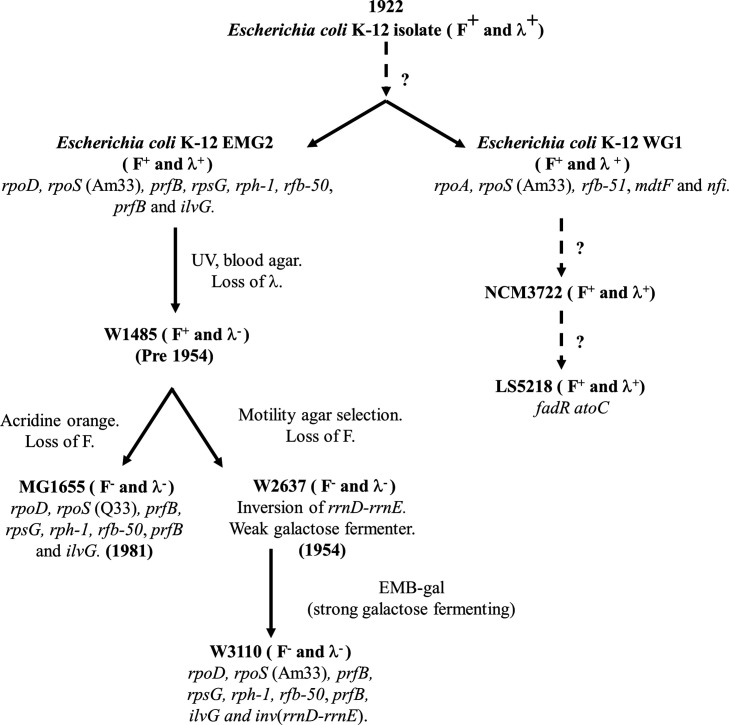
The pedigree of *E. coli* K-12 strains. The figure details the pathway of *E. coli* K-12 evolution from its isolation in 1922 to the generation of MG1655 and W3100 strains [1, 3, 4, 12]. Blood agar indicates selection on blood agar plates; UV indicates irradiation with ultraviolet light; EMB-gal indicates selection for utilization of galactose on eosin methylene blue indicator plates. Dotted lines represent uncertain evolutionary lineage events.

*Escherichia coli* K-12 was originally isolated in 1922 from a convalescent diphtheria patient and, later in the 1940s, adopted by Charles Clifton and Edward Tatum as a model organism [1–3]. Since then, *E. coli* K-12 has become the ‘workhorse’ of molecular biology, becoming arguably the premier model organism in bioscience today. MG1655 was the first *E. coli* K-12 strain to have its genome sequence published, followed by W3110, resulting in an explosion of genomic research and comparative genomics [4, 5]. However, despite its prestige, *E. coli* K-12 was stored on agar plates, stabs or slopes before cryopreservation became established, and has been repeatedly subcultured and mutagenized (Fig. 1), resulting in an organism that carries various genetic changes and has lost the ability to produce many surface-associated structures [3]. For example, *E. coli* K-12 laboratory strains are unable to synthesize O antigen on their lipopolysaccharide and no longer carry the F plasmid nor bacteriophage λ [3, 6–9]. One major strength of using *E. coli* K-12 strains for cloning and heterologous gene expression is that K-12 strains cannot establish in the human gut [10, 11] and, thus, even so-called ‘wild-type’ *E. coli* K-12 strains, like MG1655 and W3110, are very different from commensal or environmental isolates [3, 4, 12, 13].

To understand more about this important model organism, we have sequenced the genomes of two *E. coli* K-12 strains, WG1 and EMG2, the proposed ancestors of key laboratory strains (Fig. 1) [1, 2]. Isolates EMG2 (source: Clowes and Hayes) [2] and WG1 (source: J. Lederberg) are both held in the Coli Genetic Stock Center (CGSC) at Yale University (USA), labelled as the *E. coli* K-12 wild-type strain [1, 2]. However, it is unclear when each strain was deposited. For EMG2, it is specifically stated in the CGSC data entry that it does not contain any known mutations, picked up during laboratory culture, which is likely based on phenotypic analysis. WG1 is reported in the CGSC entry to be *rfb51* [8] and to potentially have a truncated F plasmid. However, it is still classified as wild-type *E. coli* K-12 in the CGSC database. The standard laboratory strains MG1655 and W3110 are both derived from strain W1485, which itself was derived directly from ‘wild-type *E. coli* K-12’ (EMG2/WG1) (Fig. 1) [1, 12]. After comparing the genomes of these ancestral strains, our analysis confirms that these strains carry genetic elements such as phage λ and the F plasmid, but indicates that they have also undergone mutational alteration during their time in laboratories.

## Methods

### Bacterial strains and whole-genome sequencing

*E. coli* K-12 strains WG1 and EMG2 were obtained for the CGSC, strain numbers CGSC 5073 and CGSC 4401, respectively [1, 2]. Each strain was sequenced using the enhanced sequencing option from MicrobesNG (https://microbesng.com/), which uses a combination of Illumina and Oxford Nanopore Technologies (ONT). Cell cultures were grown in LB medium and the cell pellet was isolated by centrifugation and resuspended in cryo-preservative in a Microbank tube (Pro-Lab Diagnostics). Approximately 2×10^9^ cells were used for high molecular mass DNA extraction using a Nanobind CCB Big DNA kit (Circulomics). DNA was quantified with the Qubit dsDNA HS assay in a Qubit 3.0 device (Invitrogen). Long-read genomic DNA libraries were prepared with the SQK-LSK109 kit (ONT) with Native Barcoding EXP-NBD104/114 (ONT), using 400–500 ng high molecular mass DNA. Twelve to twenty-four barcoded samples were pooled in a single sequencing library and loaded on a FLO-MIN106 (R.9.4 or R.9.4.1) flow cell in a GridION device (ONT). Illumina reads were adapter trimmed using Trimmomatic 0.30 with a sliding window quality cut-off of Q15 [14]. Unicycler v0.4.0 was used for genome assembly [15] and Prokka 1.11 to annotate contigs [16]. Sequence data have been deposited at GenBank/ENA/DDBJ with the accession numbers CP099590 and CP099591 for WG1, and CP099588 and CP099589 for EMG2.

### Bioinformatic analysis of genome sequences

For single nucleotide variant (SNV) calling, reads from EMG2 were aligned to the WG1 reference genome using bwa-mem and processed using SAMtools 1.2. Variants were called using VarScan with two thresholds, sensitive and specific, where the variant allele frequency is greater than 90 and 10%, respectively. The effects of variants were predicted and annotated using SnpEff. Draft genomes were visualized using Artemis [17], and comparisons between *E. coli* K-12 genomes were made using the Basic Local Alignment Search Tool (blast) from the National Center for Biotechnology Information (NCBI) (https://blast.ncbi.nlm.nih.gov/Blast.cgi), the Artemis Comparison Tool (ACT) [18] and the Proksee server (https://proksee.ca/) [19]. Genome representations were drawn using the Proksee server [19] and ACT [18]. Plasmid replicons were detected in draft genomes with PlasmidFinder 2.1 [5], using software at the Center for Genomic Epidemiology (CGE) (http://www.genomicepidemiology.org/). Insertion sequences were located using ISfinder (https://isfinder.biotoul.fr/blast.php) [20].

## RESULTS

### Comparison of the WG1 and EMG2 genomes

**Fig. 2.**
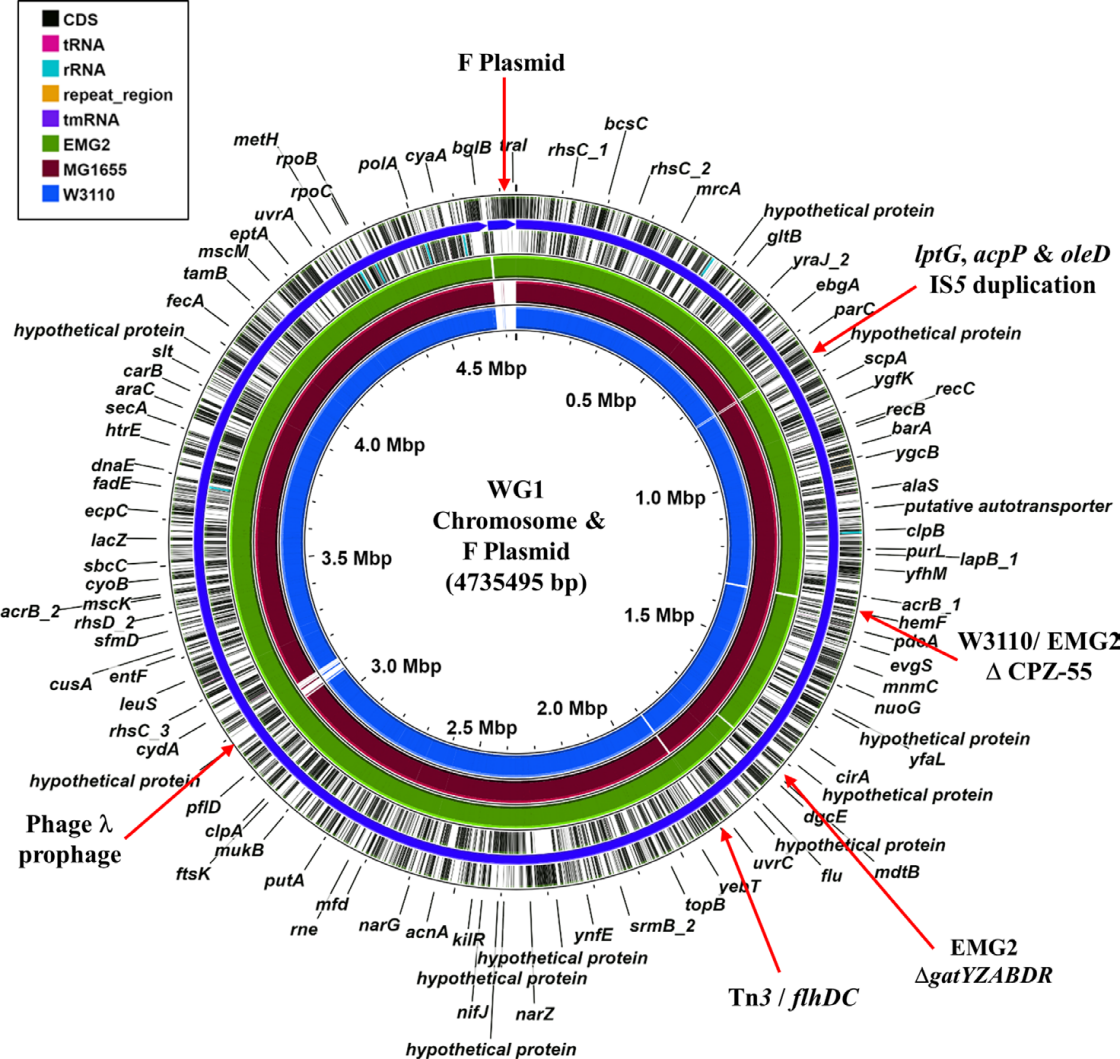
Genome comparison of different *E. coli* K-12 strains. The figure shows the comparison of the WG1 chromosome (contig 1) and F plasmid (contig 2) with the genomes of EMG2, MG1655 (NC_000913.3) and W3110 (NC_007779.1), using the Proksee server [19]. The outer two rings display the genes and features of the WG1 genome, with selected genes and differences labelled. The green, brown and blue rings illustrate the blast results when the genome sequences of *E. coli* K-12 strains EMG2, MG1655 (NC_000913.3) and W3110 (NC_007779.1), respectively, are compared to that of WG1.

**Fig. 3.**
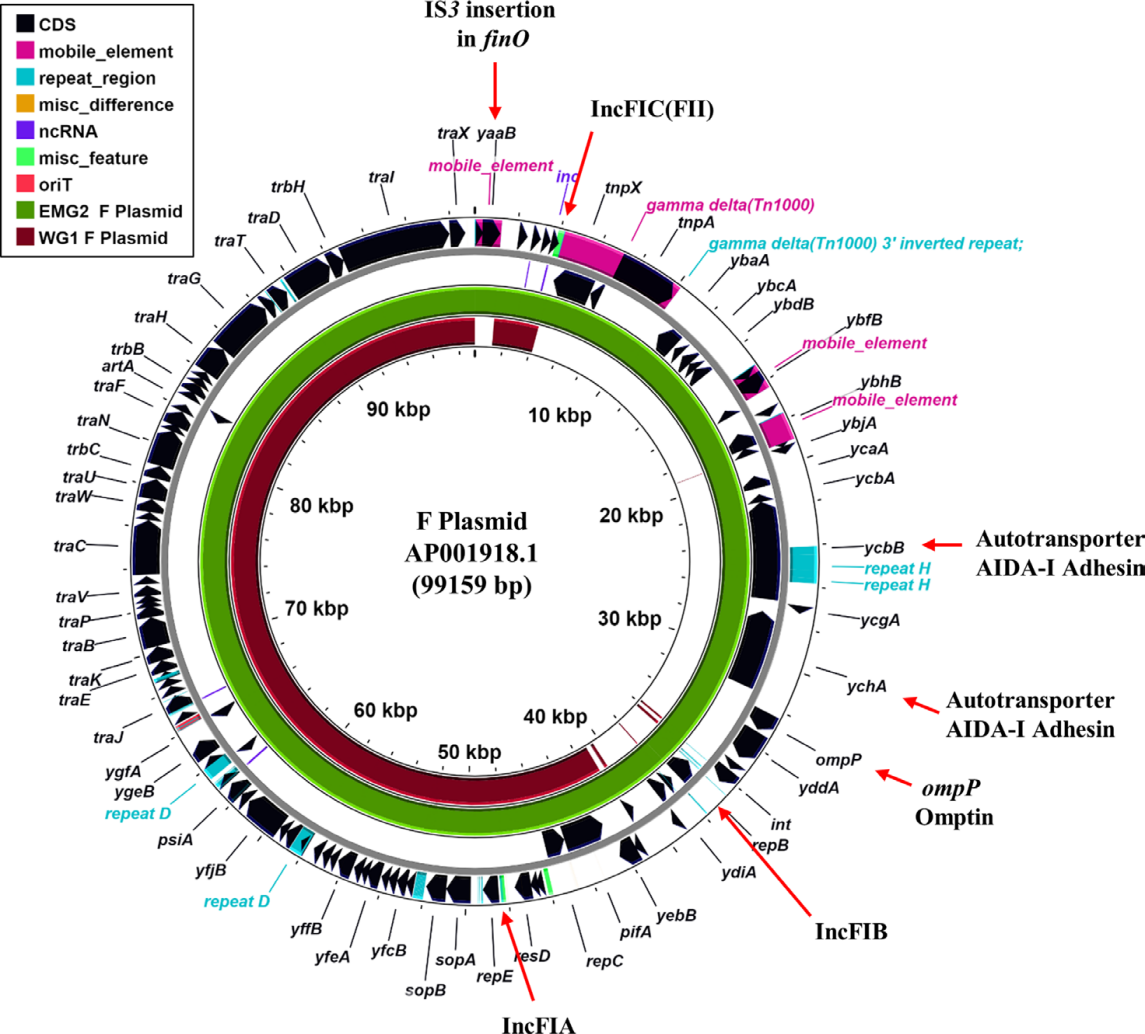
Comparison of the F plasmids from different *E. coli* K-12 strains. The figure shows the comparison of the F plasmid (AP001918.1) with that from EMG2 and WG1 using Proksee [19]. The outer two rings display the genes and features of the F plasmid, with selected genes labelled. The green and brown rings illustrate the blast results when the F plasmid sequences from EMG2 and WG1, respectively, are compared to the original F plasmid sequence.

Whole-genome sequencing of WG1 and EMG2 resulted in draft genome sequences, each comprising two contigs; the larger contig, contig 1, is the chromosomal sequence, and the smaller, contig 2, is the F plasmid (Figs 2, 3 and S1, Tables 1 and 2). Since both strains carry bacteriophage λ and the F plasmid, their genomes are slightly bigger than other sequenced *E. coli* K-12 strains, such as MG1655 and W3110 (Table 1) [4, 12]. Comparison of the genomes of both WG1 and EMG2 with those of MG1655 and W3110 indicated that, unlike W3110, no major chromosomal rearrangements had occurred in these strains (Fig. S2) [19, 21]. However, we identified a number of obvious regions of difference (Figs 2, S1 and S3). For example, both EMG2 and W3110 have lost the cryptic prophage CPZ-55, and EMG2 has lost the *gatYZABDR* locus, which is involved in galactitol metabolism [22] (Figs 2 and S4). Interestingly, the *gatYZABDR* genes appear to have been a hotspot for insertion sequence element-mediated disruption in both MG1655 and W3110, which affects expression of this region (Fig. S5) [22]. Similarly, the region upstream of *flhDC* locus, which controls flagella production, also seems to have been targeted by different transposable elements (Figs 2 and S6) [23, 24]. Note that strains that have been stored in agar stabs for many years accumulate deleterious mutations due to wholesale transposition of insertion sequences [25–27]. As insertion of different elements into this region influences motility in other *E. coli* K-12 strains, it is likely that the sequence heterogeneity found in this region produces a spectrum of effects [23, 24]. For WG1, we detected the loss of cryptic prophage CP4-6 and a large deletion of the lipopolysaccharide *O*-antigen biosynthetic cluster, previously termed *rfb-*51 (Figs S1, S3 and S4) [8]. Note that EMG2, MG1655 and W3110 carry the alternative *rfb-*50 mutation (an IS*5* disruption of the rhamnose transferase encoding gene *wbbL*), which appears to be common to most *E. coli* K-12 strains [28]. Thus, neither WG1 nor EMG2 produce O-antigen (Fig. S4) [8, 9]. Loss of O-antigen production seems to be an adaptation to laboratory life, with both the first *E. coli* strain NCTC 86 (isolated in 1885) and commonly used B strains [e.g. BL21(DE3)] all being rough in nature [13, 29, 30]. As well as these differences, WG1 also carries a block of additional genes, encoding an LPS export ABC transporter permease (*lptG*), an acyl-carrier protein (*acpP*) and a NAD-dependent epimerase/dehydratase (*oleD*), which are flanked by IS*5* elements (Figs 2 and S7).

**Table 1.** Comparison of the genomes of different *E. coli* K-12 laboratory strains

	WG1	EMG2	MG1655	W3110	NCM3722	LS5218
Accession no.	CP099590 CP099591	CP099588 CP099589	NC_000913.3	NC_007779.1	CP011495.1 CP011496.1	MVJG00000000.1
Genome size*	4 735 495 bp	4 774 480 bp	4 641 652 bp	4 646 332 bp	4 745 591 bp	4 699 198 bp
Plasmid	F plasmid	F plasmid	None	None	F plasmid	F plasmid
Total no. of CDSs†	4431	4457	4285	4213	4539	4368
G+C content	50.75 mol%	50.73 mol%	50.79 mol%	50.8 mol%	50.76 mol%	50.72 mol%

*Genome size includes the F plasmid for WG1, EMG2, NCM3722 and LS5218.

†Number of coding sequences (CDSs) is as predicted by each genome annotation.

**Table 2.** Comparison of the F plasmid from different *E. coli* K-12 laboratory strains

	WG1	EMG2	F plasmid	NCM3722	LS5218
Accession no.	CP099591	CP099589	AP001918.1	CP011496.1	CM007715.1
F plasmid size	67 408 bp	99 158 bp	99 159 bp	67 545 bp	67 502 bp
Total no. of CDSs*	73	98	105	79	83
G+C content	51.66 mol%	48.17 mol%	48.17 mol%	51.67 mol%	51.67 mol%
Plasmid replicons†	IncFIA, IncFIC(FII)	IncFIA, IncFIB, IncFIC(FII)	IncFIA, IncFIB, IncFIC(FII)	IncFIA, IncFIC(FII)	IncFIA, IncFIC(FII)

*Number of coding sequences (CDSs) is as specified by genome annotation.

†Plasmid replicons were detected using PlasmidFinder 2.1 using software at the Center for Genomic Epidemiology (CGE) [5].

### The F plasmid

Comparison with MG1655 confirmed that WG1 and EMG2 both carry the F plasmid; however, the two versions of F differ markedly in size, with that from EMG2 (99 158 bp) similar in size to the previously sequenced F plasmid (AP001918.199159 bp), whilst F from WG1 is considerable smaller (67 408 bp) (Table 2, Figs 3 and S8). This can be attributed to the loss of a large section of F in WG1, carrying the AIDA-I like autotransporter adhesin genes *ycbB* and *ychA*, the *ompP* omptin and the IncFIB replicon (Table 2, Figs 3 and S9) [7, 31]. Surprisingly, F from WG1 carries additional DNA, which includes an IncFII RepA protein (Figs S8 and S9). As in the previously sequenced F plasmid (AP001918.1), EMG2 F carries an IS*3* insertion in the *finO* gene, which leads to constitutive F transfer [7, 32, 33]. However, this insertion sequence is absent from the WG1 F (Figs 3, S8 and S9), suggesting that conjugative transfer is regulated in this plasmid and that the insertion of IS*3* must have occurred in the immediate ancestor of EMG2. Thus, it is clear that F plasmids from both EMG2 and WG1 have undergone significant laboratory-based evolution, leading to two very different plasmids.

### Bacteriophage λ

Comparison with MG1655 indicated that, as expected, both WG1 and EMG2 carry the bacteriophage λ prophage integrated between the *bioA* and *ybhC* genes (Figs 2, S1 and S10). However, comparison with the previous sequenced λ genome (NC_001416) identified some differences in λ from WG1 and EMG2, in particular with the genes encoding tail fibres J, Stf and Tfa (Fig. S11). Of note is *stf* (side tail fibre), which in λ (NC_001416) carries a frameshift disrupting the gene into two ORFs (*orf-401* and *orf-314*) [34, 35]. Bacteriophage λ carrying this lesion (λ PaPa) forms larger λ plaques [6, 35]. Thus, as *stf* remains intact in WG1 and EMG2, it is likely that both strains would produce a small plaque phenotype [6, 35].

### Similarities and differences between WG1 and EMG2

SNV calling showed that *E. coli* K-12 strains WG1 and EMG2 also differ in a number of key genes involved in important cellular functions (File S1). For example, in EMG2, the gene encoding the major sigma factor σ^70^ (*rpoD*) carries a substitution, which results in Tyr at position 571 (Fig. S12a). This is also found in MG1655 and W3110, whilst most *E. coli* strains carry His at this position. Substitutions at σ^70^ residue 571 have been shown to affect transcription at the *lac*, *araBAD*, *merT*, *merR* and the P22 phage *ant* promoters, as well as interfering with σ^70^ binding to core RNA polymerase and its ability to compete with alternative sigma factors [36–40]. Conversely, in WG1, the gene encoding the α subunit of RNA polymerase carries a mutation that results in a Gly to Arg substitution at position 311 (Fig. S12b). This alteration affects expression from both the *merT* and *merR* promoters and the anaerobically activated *pepT* promoter in *Salmonella enterica* serovar Typhimurium [38, 41]. (Note that α in *E. coli* and *S. enterica* serovar Typhimurium are identical.) As for many K-12 strains, both WG1 and EMG2 carry a truncation in *rpoS*, which encodes the stress and stationary phase sigma factor σ^S^ (Fig. S12c). (Note that the *rpoS* gene in MG1655 is the pseudo revertant *rpoS* 33Q allele [4, 12].) Additionally, *E. coli* K-12 strains also carry changes in genes that influence translation. Like MG1655 and W3110, EMG2 carries a mutation in the gene encoding release factor RF2 (*prfB*) (Thr at position 246) and a mutation in *rpsG* (30S ribosomal protein S7), which results in C-terminal extension of the S7 protein product (Fig. S12d, e). Both substitutions have been shown to affect translation, with the mutation in RF2 resulting in poor termination at UGA stop codons and the trans-translational tagging of S7 with the SsrA peptide [42–46]. Thus, it is clear that, for both EMG2 and WG1, adaptation to a laboratory lifestyle has resulted in strains with altered transcription and translation machineries, which likely impact on global gene expression.

Our analysis also identifies mutations in genes involved in metabolism and cellular homeostasis (File S1). Similar to MG1655 and W3110, EMG2 carries a frameshift in *rph* (previously termed *rph-1*) that results in a truncation of RNase PH, which affects the expression of *pyrE*, manifesting in a pyrimidine starvation phenotype (Fig. S12f) [47, 48]. Like other K-12 strains, EMG2 also carries a mutation in *ilvG*, which produces a truncated protein product that affects branch chained amino acid biosynthesis [49] (Fig. S12g). Whilst these mutations are absent from WG1, WG1 carries lesions in *mdtF* (an AcrB efflux pump homologue) and *nfi* (DNA repair endonuclease V), both of which result in truncated products (Fig. S12h, i). Thus, WG1 is likely compromised in both drug efflux and DNA damage repair [50, 51].

## Discussion

The use of *E. coli* K-12 has shaped biological knowledge and research over the last century [52]. Fred Neidhardt’s comment that ‘All cell biologists have at least two cells of interest: the one they are studying and *E. coli *’ [53] still holds true for the many scientists who have adopted *E. coli* K-12 to advance their understanding of molecular biology and microbiology. However, it is clear that adaptation to the laboratory lifestyle has resulted in *E. coli* K-12 strains that have alterations in transcription, translation, general metabolism and cellular homeostasis. Although, it is important to note that not all mutations necessarily arose as a direct consequence of laboratory growth, and it is unclear when they occurred as it is unknown when EMG2 and WG1 were deposited at the CGSC. However, in spite of this, as *E. coli* K-12 strains EMG2, MG1655 and W3110 share many common alterations (e.g. in *rpoD*, *prfB*, *rpsG*, *rph* (*rph-1*), *wbbL* (*rfb-50*), *prfB* and *ilvG*), this indicates that they share a similar lineage and that many of these mutations were fixed in their common ancestral strain (Fig. 1). However*,* WG1 carries alterations in different genes (e.g. *rfb-50*, *rpoA*, *mdtF* and *nfi*), suggesting that it is distinct from these strains (Fig. 1). It is worth noting that WG1 is similar to *E. coli* strains NCM3722 [54] and LS5218 [55]. Strain NCM3722 (CGSC 12355) was first detailed by Sydney Kustu [48] and LS5218 is an industrial strain used for the production of fatty acid derived products [55]. Both strains carry bacteriophage λ, a smaller version of the F plasmid (Tables 1 and 2, Figs S13 and S14) and contain many of the mutations carried by WG1 [54, 55].

In addition to lineage-specific mutations, it is clear that WG1 and EMG2 have undergone their own laboratory-based evolution events, such as loss of cryptic prophages and gene disruption. The suggestion is that the selection of particular traits by microbiologists has driven laboratory-based evolution. Hence, insertion sequence inactivation of *finO* in F made plasmid transfer easier to study, larger plaques enabled the intricacies of λ lysogeny to be examined and lack of O-antigen enhances plasmid transformation [6, 7, 30, 35]. Thus, our interpretation of *E. coli* biology has been inadvertently biased. Moreover, many other laboratory strains, handed down for generations, are as yet unsequenced, so it is unclear what other changes lie within those strains.

Heterogeneity in bacterial laboratory strains and plasmids has been observed many times and we are at a stage when even the same *E. coli* K-12 stock strains can produce different outcomes, calling reproducibility into question [27, 48, 56–59]. It is clear that there are significant major differences between K-12 and other commensal *E. coli* strains, and these differences became fixed in the ancestors of the very widely used MG1655 and W3110 strains. Given the different mutations seen in WG1 compared to EMG2, it seems likely that identical or similar mutations will be present in other K-12 lineages. However, due to the extensive genetic systems that have been developed, demonstration of safe use, and lack of ability to colonize humans, *E. coli* K-12 strains will justifiably continue to be widely used [10, 11, 52]. We think it is important that there is an awareness of the mutations present in K-12 strains, and the effects of these mutations on the physiology and metabolism of these strains. An understanding of the conditions that might select for mutants in laboratories, and the use of cost-effective and accurate sequencing of laboratory stocks should help to prevent further undetected mutations arising in K-12 strains, which could compromise our understanding of fundamental biological processes. Thus, it is hoped that the next century will continue to provide more insight into the complex biology and evolution of this versatile organism. Indeed, appreciation of various K-12 strains, as well differences between various bacterial families, is sure to enhance our understanding of life.

## Supplementary Data

Supplementary material 1

Supplementary material 2
